# The Effect of Parental Faecal Microbiome Transplantation from Children with Autism Spectrum Disorder on Behavior and Gastrointestinal Manifestations in the Male Offspring of Shank3 Mice

**DOI:** 10.3390/ijms26135927

**Published:** 2025-06-20

**Authors:** Veronika Borbélyová, Jakub Szabó, Petronela Sušienková, Judith Potvin, Paulína Belvončíková, Tim Groß, Alžbeta Jančovičová, Zuzana Bačová, Barbara Rašková, Ivan Szadvári, Matúš Antal, Zdenko Pirník, Miloslav Karhánek, Katarína Šoltys, Roman Gardlík, Peter Celec, Daniela Ostatníková, Aleksandra Tomova

**Affiliations:** 1 Institute of Molecular Biomedicine, Faculty of Medicine, Comenius University, Sasinkova 4, 811 08 Bratislava, Slovakia; veronika.borbelyova@fmed.uniba.sk (V.B.); jakub.szabo@fmed.uniba.sk (J.S.); petronela.susienkova@imbm.sk (P.S.); belvoncikova2@uniba.sk (P.B.); gross.tim98@gmail.com (T.G.); alzbeta.jancovicova@savba.sk (A.J.); roman.gardlik@fmed.uniba.sk (R.G.); peter.celec@fmed.uniba.sk (P.C.); 2 Institute of Medical Physics and Biophysics, Faculty of Medicine, Comenius University, Sasinkova 2, 813 72 Bratislava, Slovakia; 3 Department of Surgery, Kreisklinik Wörth a.d. Donau, Krankenhausstraße 2, 930 86 Wörth an der Donau, Germany; 4 Institute of Experimental Endocrinology, Biomedical Research Center, Slovak Academy of Sciences, Dúbravská cesta 9, 845 05 Bratislava, Slovakia; zuzana.bacova@savba.sk (Z.B.); antal28@uniba.sk (M.A.); zdenko.pirnik@fmed.uniba.sk (Z.P.); miloslav.karhanek@savba.sk (M.K.); 5 Institute of Physiology, Faculty of Medicine, Comenius University, Sasinkova 2, 813 72 Bratislava, Slovakia; barbara.raskova@fmed.uniba.sk (B.R.); ivan.szadvari@fmed.uniba.sk (I.S.); daniela.ostatnikova@fmed.uniba.sk (D.O.); 6 Institute of Organic Chemistry and Biochemistry of the Czech Academy of Sciences, Fleming pl. 542/2, 160 00 Prague, Czech Republic; 7 Comenius University Science Park, Comenius University, 841 04 Bratislava, Slovakia; katarina.soltys@uniba.sk; 8 Institute of Pathophysiology, Faculty of Medicine, Comenius University, Sasinkova 4, 811 02 Bratislava, Slovakia

**Keywords:** neurodevelopmental disorder, SHANK 3 gene, maternal microbiota, fecal microbiota transplantation, behavior

## Abstract

The increasing incidence of autism spectrum disorder (ASD) increases the urgency of establishing the mechanism of its development for effective prevention and treatment. ASD’s etiology includes genetic predisposition and environmental triggers, both of which can play a role in the changed microbiota. Recent research has proved the impact of maternal microbiota on the neurodevelopment of the child. To investigate the co-play of genetic and microbiota factors in ASD development, we performed fecal microbiota transplantation (FMT) from children with ASD to female *Shank3b^+/−^* mice and studied the autism-like symptoms in the male *Shank3b^−/−^* and wild-type (WT) offspring. WT animals with prenatal exposure to ASD microbiota had delayed neurodevelopment and impaired food intake behavior, but also elevated plasma leptin concentration and body weight. *Shank3b^−/−^* mice after FMT ASD exhibited impaired learning and exacerbated anxiety-like behavior in adulthood. Interestingly, FMT ASD improved learning in adolescent *Shank3b^−/−^* mice. Prenatal exposure to ASD microbiota decreased the activity of hypocretin neurons of the lateral hypothalamic area in both genotypes. The combination of genetic predisposition and FMT ASD led to an increased colon permeability, evaluated by zonula occludens (ZO1, ZO3) and claudin factors. These results suggest the effect of parental FMT exposure on shaping offspring behavior in *Shank3b^−/−^* mice and the potential of microbiota in the modulation of ASD.

## 1. Introduction

Autism spectrum disorder (ASD) is a heterogeneous group of disorders characterized by persistent deficits in reciprocal social interaction and social communication, and by a range of restricted, repetitive, and inflexible patterns of behavior and interests. Boys are diagnosed with ASD four times more often than girls [1]. Although ASD exhibits high heritability, the interaction of genetic and environmental factors plays a crucial role in its development [2,3]. Advancing research over the past years has enriched our knowledge about the microbiota–gut–brain axis [4,5], suggesting that gut microbiota could contribute to triggering ASD manifestations on the existing genetic background. Firstly, it has been shown that variations in the gut microbiota composition are associated with changes in the normal functioning of the nervous system and modulate many aspects of behavior, including ASD symptoms [2,6,7,8,9]. Secondly, most children with ASD experience gastrointestinal (GI) disorders such as constipation, diarrhea, and nutrition issues [10,11,12], which can reasonably be attributed to changes in their gut microbiota. Indeed, recent metagenomic studies have confirmed significant differences in the gut microbiota of ASD children compared to neurotypical children, particularly at the phyla level (Bacteroidetes/Firmicutes ratio), and at the species level (some *Clostridium*, *Desulfovibrio, Lactobacillus*, etc.) [13,14,15]. Described hypotheses of ASD include the etiopathogenetic role of specific bacteria, such as *Desulfovibrio* and *Clostridia* [16,17]. Some bacteria are found only in the gut microbiota of ASD children or only in matching controls, while other bacteria are found in significantly higher or lower abundance [18,19,20]. Although the role of gut microbiota in ASD pathogenesis is not yet completely understood, preclinical studies suggest its role in ASD-like behavior in germ-free mice observed after fecal microbiota transplantation (FMT) from people with ASD [21].

Dysbiosis found in people with ASD might be the reason for disturbed intestinal permeability and a low degree of intestinal inflammation [22]. “Leaky gut” appears to be the cause of frequent GI symptoms in these children [23,24,25,26], which also affects their behavior [27]. Children with ASD have not only a higher rate of GI disorders than neurotypical controls, but they also frequently display unusual feeding behaviors, such as food selectivity [28,29,30], associated with the severity of ASD symptoms [10,31]. Since diet and eating habits affect gut microbiota composition [32], it is not surprising that “picky eaters” with ASD display different gut microbiota [31]. Microbial metabolites and gut peptides are involved in the homeostatic control of food intake through the hypothalamic and brainstem structures [33,34], which emphasize the role of the gut–brain axis in central food intake regulation. Ingestive behavior is regulated by the integrative system of the brain, which involves both homeostatic and hedonic neural circuits [35].

Genetic factors play a considerable role in the development of ASD. The Simons Foundation Autism Research Initiative (SFARI) Gene Database is continuously updated to provide comprehensive information on all known human genes associated with ASD (https://gene.sfari.org/). Among those strongly implicated in ASD pathology have been SHANK genes, which represent postsynaptic scaffolding proteins [36]. Mutations in SHANK genes have been associated with ASD as well as with intellectual disability [36,37]. Particularly, SHANK3 plays a crucial role in cytoskeletal stability, and its deficiency has been linked to impaired neuritogenesis and synaptogenesis [38,39], processes that are associated with the etiology of ASD. *Shank3*-deficient mice exhibit ASD-like behaviors [40,41] with varying degrees of severity [42,43,44], making them a widely accepted animal model for studying ASD [45].

Prenatal neurodevelopment and intensive early postnatal development of the nervous system, including the brain, are crucial periods for ASD development. During pregnancy, the fetus is susceptible to prenatal risk factors associated with ASD, including genetic predisposition, epigenetic modifications induced by bacterial products, mitochondrial dysfunction, hormonal imbalances, oxidative stress, inflammation, and the effects of postbiotics derived from an altered gut microbiome [46,47,48,49].

Therefore, growing evidence supports a multifactorial etiology of ASD, including a complex interplay between genetic susceptibility and environmental factors, such as the gut microbiota. In the present study, we investigated this interaction by applying fecal microbiota from children with ASD and neurotypical controls prenatally to mice with and without genetic predisposition for ASD (*Shank3b^−/−^*, WT), aiming to explore their combined effect on ASD-like symptoms in the mouse offspring. Because autism spectrum disorder (ASD) is diagnosed in boys about four times more often than in girls [50], we decided to test our hypotheses initially in male animals.

## 2. Results

### 2.1. Behavior

#### 2.1.1. Neurodevelopmental Milestones

**Figure 1:**
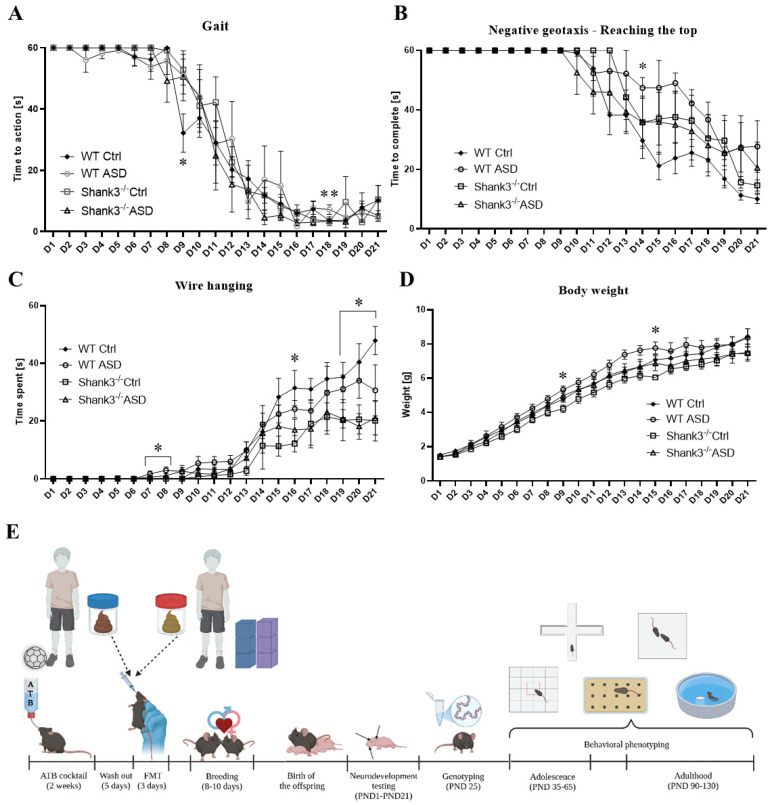
Neurodevelopmental milestones. (**A**) Gait (* =WT ASD > WT Ctrl; ** = WT ASD > WT Ctrl). (**B**) Proprioception and vestibular function (* = WT ASD < all). (**C**) Neuromuscular apparatus using wire hanging (D7-8, * =WT ASD > WT Ctrl; D16, D19-21, * = WT ASD < WT Ctrl). (**D**) Body weight (D9, D15, * = WT ASD > WT Ctrl). (**E**) Experimental design. Created by BioRender.com. PND/D—postnatal day, ATB—antibiotic cocktail, FMT—fecal microbiota transplantation, ASD–mice prenatally exposed to FMT from children with ASD, Ctrl—mice prenatally exposed to FMT from neurotypical children, mean ± SEM, * *p* < 0.05, ** *p* < 0.01, WT Ctrl, *n* = 11; WT ASD, *n* = 5; Shank3 Ctrl, *n* = 7; and Shank3 ASD, *n* = 6.

Testing neurodevelopmental milestones from postnatal day (PND) 1 to PND 21, the effect of FMT ASD was almost exclusively observed in the WT group. The FMT received from ASD patients postponed the development of stable gait in WT mice, compared to those who received FMT from neurotypical (NT) controls (CTRL) (*p* < 0.05, Figure 1A). FMT ASD further postponed the development of proprioceptive and vestibular functions in WT animals, as compared to the rest of the groups (*p* < 0.05, Figure 1B). Conversely, in the development of neuromuscular apparatus, FMT ASD allowed WT mice to outperform the other groups during early development (PND7-8, *p* < 0.05), but their developmental progress stagnated during later days (D16, D19-D21, *p* < 0.05, Figure 1C) in comparison to WT mice that received FMT from NT controls. Finally, WT mice prenatally exposed to FMT ASD showed increased developmental body weight on selected days (PND9, PND15, *p* < 0.05, Figure 1D).

Besides the main findings, several parameters presented with statistically significant effects likely attributable to methodological variability within the final days of neurodevelopmental testing. In the walking task, the groups differed in time required to pass the test at PND16 [F(3,26) = 2.8, *p* < 0.05], PND18 [F(3,26) = 5.7, *p* < 0.01], and PND20 [F(3,26) = 4.1, *p* < 0.05, Figure S1A]. At PND16, this effect was only nominal and did not translate into significant between-group effects in post hoc comparison. At PND18, the effect comprised WT ASD mice, which required a longer time to perform the task (27.2 ± 9.8 s, *p* < 0.01) than the rest of the groups (WT CTRL: 7.3 ± 2.1 s, *Shank3^−/−^* CTRL: 5.8 ± 1.6 s, *Shank3^−/−^* ASD: 6.2 ± 1.4 s). At PND20, the effect was fully explained by the *Shank3^−/−^* CTRL mice, which required the least time to complete the task (6.5 ± 2.1 s, *p* < 0.05) among all groups (WT CTRL: 10.1 ± 4.7 s, WT ASD: 36.5 ± 12.9 s, *Shank3^−/−^* ASD: 12.2 ± 3 s). Additionally, no significant differences were observed between the groups in the first time to perform the walk milestone [F(3,26) = 1.3, *p* = 0.28, Figure S1B]. In surface righting, the animal groups differed in time required to complete the test at PND10 [F(3,26) = 4.1, *p* < 0.05, Figure S1C]. The Bonferroni-corrected post hoc test showed that this effect was caused exclusively by the *Shank3^−/−^* CTRL group, which required the most time to pass the test (4.1 ± 1.1 s, *p* < 0.05) among all the groups (WT CTRL: 1.3 ± 0.2 s, WT ASD: 1.5 ± 0.6 s, *Shank3^−/−^* ASD: 1.7 ± 0.5 s). No statistical differences between the groups were observed in the first time to pass the surface righting [F(3,26) = 0.85, *p* = 0.48, Figure S1D]. The time required to turn within the negative geotaxis task presented with high variability, resulting in no statistical difference among the groups at any PND (*p* > 0.05, Figure S1E). Similarly, no group differences were observed in the first time to turn successfully within the negative geotaxis task [F(3,26) = 2.4, *p* = 0.09, Figure S1F].

No significant differences between the groups were observed in the first time the ears of the animals unfolded [F(3,26) = 1.3, *p* = 0.28, Figure S2A], their ears twitched [F(3,26) = 0.53, *p* = 0.67, Figure S2B], in the time the animals were able to right themselves in the air [F(3,26) = 1.8, *p* = 0.18, Figure S2C], their incisors erupted [F(3,26) = 1.7, *p* = 0.2, Figure S2D], they were able to grasp with their front paws [F(3,26) = 2.7, *p* = 0.06, Figure S2E], or the time to adjust the front paws to a perpendicular surface [F(3,26) = 2.1, *p* = 0.12, Figure S2F], nor when the auditory [F(3,26) = 0.99, *p* = 0.41, Figure S2G], or tactile startle reflex developed [F(3,26) = 2.1, *p* = 0.13, Figure S2H]. However, the groups differed in the first time their eyes opened [F(3,26) = 5.2, *p* < 0.01, Figure S2I]. This effect was explained by the WT ASD group, which opened their eyes earlier (12.2 ± 0.4 PND, *p* < 0.05) than the WT CTRL group (13.5 ± 0.3 PND).

#### 2.1.2. Behavioral Testing in Adolescence

**Figure 2:**
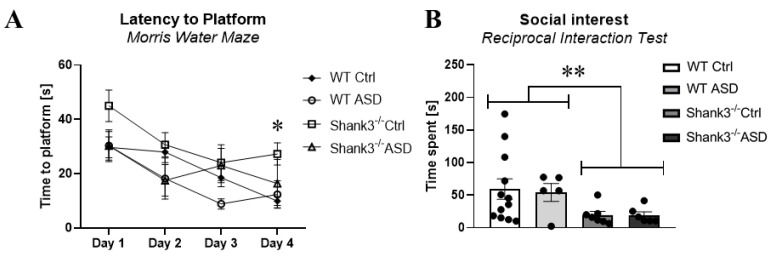
Behavioral testing during adolescence. (**A**) The training phase of memory and learning testing (* = *Shank3^−/−^* ASD < *Shank3^−/−^* Ctrl). (**B**) Social interest in sociability testing (** = *Shank3^−/−^* < WT). ASD—mice prenatally exposed to fecal microbiota transplantation (FMT) from children with ASD, Ctrl—mice prenatally exposed to FMT from neurotypical children, mean ± SEM, * *p* < 0.05, ** *p* < 0.01, WT Ctrl, *n* = 11; WT ASD, *n* = 5; *Shank3^−/−^* Ctrl, *n* = 7; and Shank3 ASD, *n* = 6.

The effects of FMT and its interaction with mouse genotypes were most limited in animal behavior during adolescence. We observed a beneficial effect of FMT from ASD patients during the training sessions when evaluating learning and memory. The *Shank3^−/−^* mice exposed to FMT ASD showed faster escape latency on the fourth training day of the MWM as compared to the *Shank3^−/−^* exposed to FMT from NT controls (*p* < 0.05, Figure 2A). In contrast, we observed a dominant effect of genotype in social behavior, regardless of the FMT exposure. The *Shank3^−/−^* mice spent 50% less time interacting with socially unfamiliar mice than WT mice (*p* < 0.01, Figure 2B). We did not observe any other statistically significant effects in behavior during adolescence.

Besides the main findings from the behavioral testing in adolescence, the rest of the behavioral parameters showed no difference between the groups. These included the analysis of locomotor activity [F(3,26) = 2.6, *p* = 0.07, Figure S3A], explorative (rearing) behavior [F(3,26) = 1.6, *p* = 0.22, Figure S3B], repetitive grooming behavior [F(3,26) = 4.1, *p* = 0.16, Figure S3C], anxiety-like behavior measured via time spent in the center zone of the open field test [F(3,26) = 2.4, *p* = 0.09, Figure S3D], as well as measured via time spent in open arms of the elevated plus maze [F(3,26) = 0.96, *p* = 0.42, Figure S3E], marble burying [F(3,26) = 1.5, *p* = 0.24, Figure S3F], social disinterest [F(3,26) = 0.31, *p* = 0.82, Figure S3G], learning [F(3,26) = 1.6, *p* = 0.21, Figure S3H], and cognitive flexibility [F(3,26) = 2.26, *p* = 0.11, Figure S3I]. The only additional behavioral parameter which produced a significant group difference in adolescence was the training part of the reversal learning phase of the Morris water maze. This difference was observable on day 1 [F(3,26) = 6.3, *p* < 0.01] and on day 2 [F(3,26) = 3.2, *p* < 0.05, Figure S3G]. On day 1, the effect was explained by the *Shank3^−/−^* CTRL mice, which exhibited the longest time locating the hidden platform (50.4 ± 3.1 s, *p* < 0.01) among the rest of the groups (WT CTRL: 34.7 ± 3.4 s, WT ASD: 26.8 ± 4 s, *Shank3^−/−^* ASD: 31.4 ± 4.2 s). On day 2, the effect was explained by the WT ASD mice, which showed the shortest time locating the hidden platform (13.7 ± 4.7 s, *p* < 0.05) of all the groups (WT CTRL: 34.9 ± 5 s, *Shank3^−/−^* CTRL: 39.7 ± 5.4 s, *Shank3^−/−^* ASD: 31.3 ± 5.9 s).

#### 2.1.3. Behavioral Testing in Adulthood

**Figure 3:**
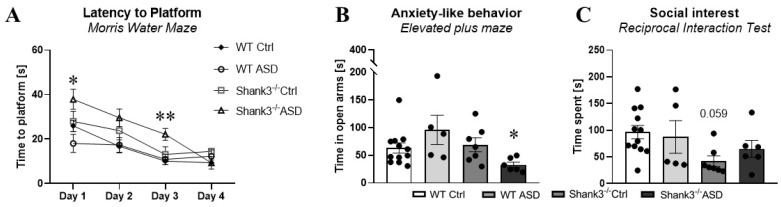
Behavioral testing in adulthood. (**A**) The training phase of memory and learning testing (D1, D3, * = *Shank3^−/−^* ASD > *Shank3^−/−^* Ctrl). (**B**) Anxiety-like behavior as measured in the elevated maze plus shaped test (* = *Shank3^−/−^* ASD > *Shank3^−/−^* Ctrl). (**C**) Social interest in sociability testing. ASD—mice prenatally exposed to fecal microbiota transplantation (FMT) from children with ASD, Ctrl—mice prenatally exposed to FMT from neurotypical children. D = postnatal day, mean ± SEM, * *p* < 0.05, ** *p* < 0.01, WT Ctrl, *n* = 11; WT ASD, *n* = 5; *Shank3^−/−^* Ctrl, *n* = 7; and Shank3 ASD, *n* = 6.

In contrast to the result demonstrating a potential beneficial impact of FMT from ASD patients in a group of *Shank3^−/−^* mice in adolescence, in adulthood the effect was rather opposite. In the training phase of learning and memory testing, *Shank3^−/−^* mice prenatally exposed to FMT ASD showed the worst learning curve among the groups, which manifested in a statistically significant difference in comparison to the Shank3^−/−^ mice prenatally exposed to FMT from NT controls (day 3, *p* < 0.01, Figure 3A). The adverse effect of FMT ASD was further demonstrated in *Shank3^−/−^* mice by the shortest time spent in the open arms of the EPM, corresponding to the most prominent anxiety-like behavior among the murine groups. The *Shank3^−/−^* prenatally exposed to FMT ASD spent twice as much time in the open arms than the *Shank3^−/−^* mice prenatally exposed to FMT from NT children (*p* < 0.05, Figure 3B). No social deficits similar to those observed in adolescence were recorded in adulthood. The difference between *Shank3^−/−^* mice and WT controls only approached the level of statistical significance (*p* = 0.059, Figure 3C). Nevertheless, a degree of data clustering can be observed within the WT animals exposed to FMT ASD prenatally. However, the extent to which this phenomenon is an effect of the FMT itself needs to be further investigated.

Beside the main findings from the behavioral testing in adulthood, the rest of the behavioral parameters showed no difference between the groups. These included the analysis of locomotor activity [F(3,26) = 2.2, *p* = 0.1, Figure S4A], explorative behavior [F(3,26) = 2.3, *p* = 0.09, Figure S4B], repetitive behavior [F(3,26) = 4.1, *p* = 0.17, Figure S4C], anxiety-like behavior measured via the time spent in the center zone of the open field test [F(3,26) = 1.5, *p* = 0.24, Figure S4D], marble burying [F(3,26) = 0.77, *p* = 0.52, Figure S4E], social disinterest [F(3,26) = 1.6, *p* = 0.23, Figure S4F], learning [F(3,26) = 2.5, *p* = 0.08, Figure S4G], and cognitive flexibility [F(3,26) = 1.64, *p* = 0.21, Figure S4H]. Similarly to the behavioral testing during adolescence, the only additional behavioral parameter which produced a significant group difference in adulthood was the training part of the reversal learning phase of the Morris water maze. Once again, we observed a similar finding—the groups differed only on day 1 [F(3,26) = 3.3, *p* < 0.05] and day 2 of the training phase [F(3,26) = 2.9, *p* < 0.05, Figure S4I]. Importantly though, neither of these effects were large enough to produce a between-group difference in a Bonferroni-corrected post hoc analysis.

### 2.2. Food Intake and Preference Studies

**Figure 4:**
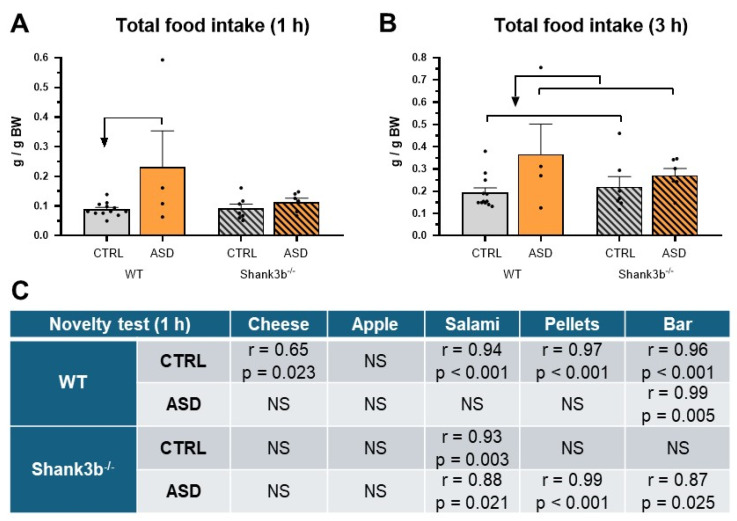
Total food intake after 1 h (**A**) and after 3 h (**B**) during the Novelty test and correlation between the intake (g) and preference (%) of all individual tested compounds during the first hour (**C**) in the overnight fasted *Shank3b* genotype mice prenatally exposed to gut microbiota from children with neurotypical development (CTRL, *n* = 19) and children with autism spectrum disorder (ASD, *n* = 10), respectively, mean ± SEM. The arrow indicates a statistically significant difference from the respective group (*p* < 0.05). r 0.36 to 0.67 indicates a moderate correlation, r = 0.68 to 0.89 a strong correlation, and r > 0.90 a very strong correlation. NS—not significant.

No significant effect of the *Shank3b* genotype or prenatal exposure to FMT was found in male mice during the 24 h period of voluntary total food intake or preference in the sweet solution test (F1,25 = 0.228, *p* = 0.637 for genotype, F1,25 = 0.086, *p* = 0.772 for FMT and F1,25 = 0.318, *p* = 0.578 for genotype, F1,25 = 0.646, *p* = 0.429 for FMT) and sweet–fat solution test (F1,25 = 1.047, *p* = 0.316 for genotype, F1,25 = 0.735, *p* = 0.399 for FMT and F1,25 = 0.057, *p* = 0.814 for genotype, F1,25 = 0.053, *p* = 0.819 for FMT). However, in overnight-fasted male mice, a significant effect of prenatal exposure to FMT in the Novelty test was found on total food intake during the first hour (F1,25 = 5.261, *p* = 0.03, Figure 4A) and during the 3 h period (F1,25 = 4.859, *p* = 0.037, Figure 4B). While the total food intake was significantly higher during the first hour only in WT mice prenatally exposed to FMT ASD compared to those exposed to FMT CTRL microbiota (Figure 4A), in the later time interval, prenatal exposure to FMT ASD significantly increased total food intake, irrespective of *Shank3b* genotype (Figure 4B). In WT mice prenatally exposed to FMT ASD, correlations between intake of the studied food components during the first hour and their preference in the Novelty test were significantly impaired compared to the mice prenatally exposed to FMT CTRL (Figure 4C). Within the *Shank3b^−/−^* genotype, the correlations were already significantly disrupted in the mice prenatally exposed to FMT CTRL while the FMT ASD group paradoxically showed higher level of correlations (Figure 4C).

**Figure 5:**
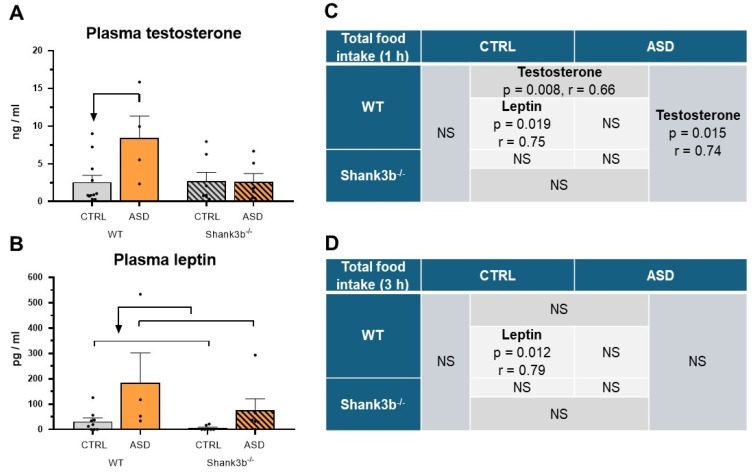
Plasma testosterone concentrations (**A**), plasma leptin concentrations (**B**) and correlation analysis between the plasma testosterone and plasma leptin concentrations with total food intake during the first hour (**C**) and during the 3 h period (**D**) in the Novelty test in the overnight fasted male *Shank3b* genotype mice prenatally exposed to gut microbiota from children with neurotypical development (CTRL) and from children with autism spectrum disorder (ASD), mean ± SEM. The arrow indicates a statistically significant difference from the respective group (*p* < 0.05). r = 0.36 to 0.67 indicates a moderate correlation, r = 0.68 to 0.89 a strong correlation, and r > 0.90 a very strong correlation. NS—not significant.

A significant effect of prenatal exposure to FMT on plasma testosterone concentrations (F1,24 = 4.294, *p* = 0.049, Figure 5A) and plasma leptin concentrations (F1,22 = 6.881, *p* = 0.016, Figure 5B) was found. In addition, a significant effect of prenatal exposure to FMT with *Shank3b* genotype interaction on plasma testosterone concentrations was also found (F1,24 = 4.52, *p* = 0.044, Figure 5A). While prenatal exposure to FMT ASD increased plasma concentrations of testosterone more than 3 times compared to those exposed to FMT CTRL in WT (Figure 5A), the same prenatal exposure to FMT ASD increased plasma leptin concentrations more than 5 times irrespective to *Shank3b^−/−^* mice (Figure 5B). A significant correlation between plasma testosterone concentrations and total food intake during the first hour in the Novelty test was found in all WT mice and in all mice prenatally exposed to ASD microbiota (Figure 5C), but the correlation with plasma leptin concentrations was significant only in prenatally FMT CTRL exposed WT mice (Figure 5C,D).

**Figure 6:**
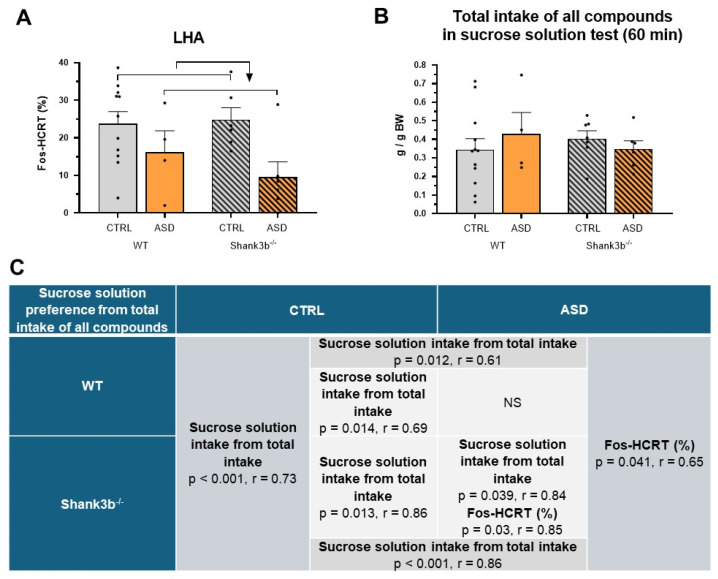
The percentage of Fos-HCRT immunopositive neurons in the lateral hypothalamic area (LHA) (**A**), total intake of all compounds (**B**), and correlation analysis between the individual sucrose solution preference and its intake from total intake of all compounds as well as between the individual sucrose solution preferences from total intake of all compounds and percentage of Fos-HCRT neurons in LHA (**C**) during the 60 min-lasting sweet solution test in the overnight fasted male *Shank3b* genotype mice prenatally exposed to gut microbiota from neurotypical control children (CTRL) and from children with autism spectrum disorder (ASD), mean ± SEM. The arrow indicates a statistically significant difference from the respective group (*p* < 0.05). r 0.36 to 0.67 indicates a moderate correlation, r 0.68 to 0.89 a strong correlation, and r > 0.90 a very strong correlation. NS—not significant.

**Figure 7:**
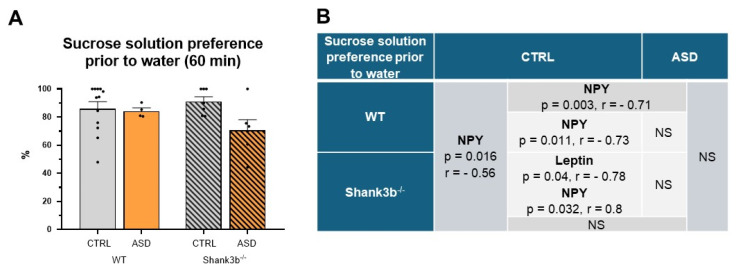
Sucrose solution preference prior to water (**A**) and its correlation analysis between plasma testosterone, leptin, and NPY level (**B**) during the 60 min-lasting sweet solution test in overnight fasted male *Shank3b* genotype mice prenatally exposed to gut microbiota from children with neurotypical development (CTRL) and from children with autism spectrum disorder (ASD), mean ± SEM. The arrow indicates a statistically significant difference from the respective group (*p* < 0.05). r = 0.36 to 0.67 indicates a moderate correlation, r = 0.68 to 0.89 is a strong correlation, and r > 0.90 a very strong correlation. NS—not significant.

Our results show a significant effect of prenatal exposure to FMT on the phasic activity of hypocretin (HCRT) neurons in the lateral hypothalamic area (LHA) (F1,23 = 7.626, *p* = 0.011, Figure 6A). Only prenatal exposure to ASD microbiota decreased the percentage of Fos-activated HCRT LHA neurons, irrespective of *Shank3b* genotype (Figure 6A), in overnight-fasted male mice in the 60 min-lasting sweet solution test. The number of HCRT immunopositive LHA neurons (87.7 ± 1.7) did not statistically differ between studied groups. Male mice did not differ in studied parameters during the 60 min-lasting sweet solution test (except for intake of sucrose compared to water intake in ASD groups, *p* < 0.05), and no statistically significant effect was seen in overnight fasted male mice in the total intake of all compounds (Figure 7B) and sucrose solution preference (Figure 6A). The close correlation between the preference and intake of sucrose solution from the total intake of all compounds in the 60 min-lasting sucrose solution test was lost in WT male mice prenatally exposed to FMT ASD (Figure 6C). Unlike prenatally FMT CTRL male mice, a positive correlation between the percentage of Fos-HCRT neurons in LHA and sucrose solution preference from total intake of all compounds was found in mice prenatally exposed to FMT ASD (Figure 6C), especially in *Shank3b^−/−^* ones (Figure 6C).

Although no correlation was found between plasma levels of testosterone, leptin, or NPY with the total intake of all compounds during the 60 min-lasting sweet solution test, a significant positive correlation between sucrose solution preference and plasma NPY level was found in animals prenatally exposed FMT CTRL (Figure 7B). The plasma levels of NPY did not differ between any studied groups of male mice. The results suggest dysregulation of the homeostatic or hedonic pathway of food intake regulation by prenatal exposure to ASD microbiota partially modified by the *Shank3b* genotype.

### 2.3. Microbiota

**Figure 8:**
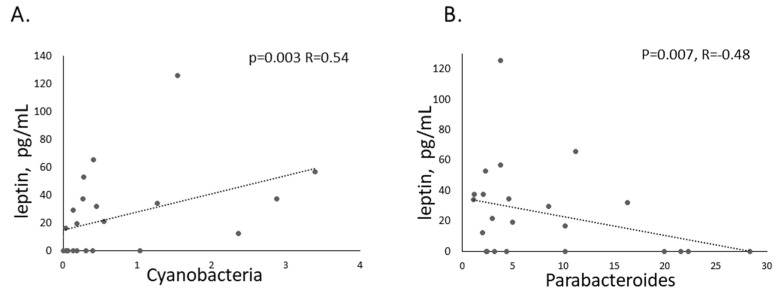
The correlation of relative abundance of (**A**). *Cyanobacteria* and (**B**). *Parabacteroides* with leptin plasma concentrations in offspring of animals exposed to fecal microbial transplantation.

In order to ensure the mother’s microbiota has influence on offspring, breeding of the exposed females with unexposed males occurred one day after FMT. The bacterial composition of donors’ microbiota was evaluated using RT-PCR (primers listed in the Table S1). In the pooled ASD donor sample, there was an increased abundance of *Prevotella* (CFU/g stool) and a decreased Firmicutes/Bacteroidetes ratio. In contrast, the pooled CTL donor sample showed an increased count of *Lactobacillus*. These patterns were also observed in the corresponding offspring compared to animals that did not receive FMT, suggesting that the maternal FMT influenced the offspring’s microbiome. Microbiota establishment during behavioral testing and the measurement of biological markers were performed using 16sRNA sequencing. A pairwise comparison of the colon microbiota from experimental animals found that *Shank3b^−/−^* mice prenatally exposed to FMT ASD had decreased alpha diversity compared to the WT CTRL group at the phyla level (Shannon, *p* = 0.027, Simpson *p* = 0.02, post hoc Mann–Whitney/Kruskal–Wallis test). Although beta-diversity (PCoA, NMDS) was not significantly different between groups, some taxa had different abundance (*p* < 0.05). The phylum Cyanobacteria was significantly lower in *Shank3b^−/−^* animals compared to the WT mice. Its abundance also positively correlated with plasma concentrations of leptin (Figure 8A). The differences between the microbiota after prenatal FMT CTRL and FMT ASD at the genus level were limited: for example, WT CTRL had a higher abundance of *Parabacteroides*, *Dubosiella,* and *Eubacterium* and WT ASD had an increased abundance of *Intestinimonas*. All *Shank3b^−/−^* animals had more abundant *Desulfovibrio*, *Lactobacillus*, *Lachnospiraceae*, *Parabacteroides*, *Tyzzerella* and *Turicibacter* and less abundant *Roseburia*, *Butyricicoccus*, *Alloprevotella*, and *Colidextribacter* compared to corresponding WT groups. The negative correlation of *Parabacteroides* with leptin concentrations is present in Figure 8B. Bacterial genera such as *Blautia*, *Lachnoclostridium*, *Butyricicoccus*, *Roseburia*, *Intestinimonas* and *Desulfovibrio* correlated with plasma testosterone levels in mice after the exposure to FMT ASD.

### 2.4. Permeability of the Gut

**Figure 9:**
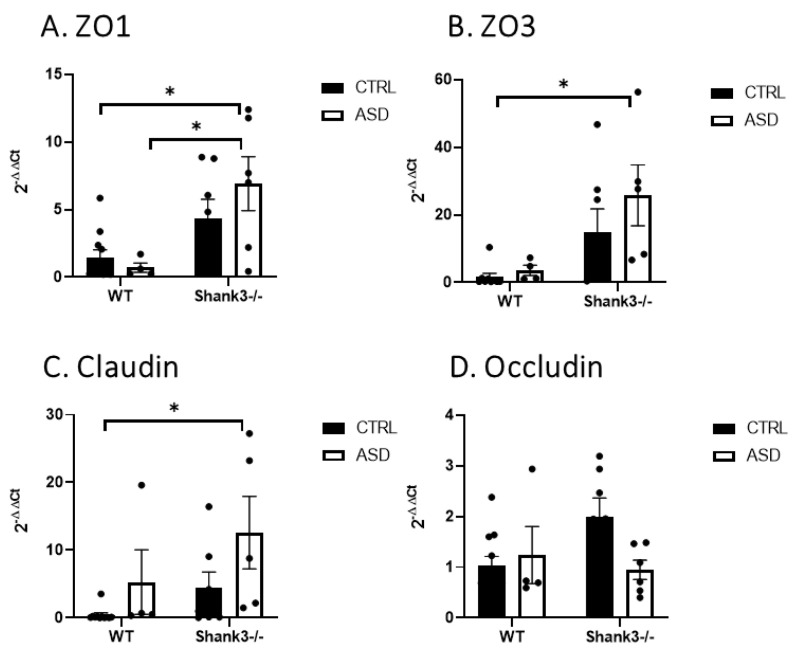
Markers of intestinal permeability in mice after prenatal exposure to FMT from boys with ASD and neurotypical controls (CTRL). (**A**) Zonula occludens -1 (ZO1). (**B**) Zonula occludens-3 (ZO3). (**C**) Claudin. (**D**) Occludin, mean ± SEM, * *p* < 0,05,WT Ctrl, *n* = 11; WT ASD, *n* = 4; *Shank3^−/−^* Ctrl, *n* = 7; and *Shank3^−/−^* ASD, *n* = 6.

Analyzing the prenatal impact of FMT on the intestines of Shank3b mice, we found that three out of four gut permeability markers, zonula occludens ZO1, ZO3, and claudin, were significantly higher in the colon of *Shank3b^−/−^* offspring animals exposed to transfer of microbiota from children with ASD compared to WT mice prenatally exposed to the control microbiota (Figure 9). Also, ZO1 demonstrated increased intestinal permeability in *Shank3b^−/−^* versus WT animals in the group exposed to FMT ASD (Figure 9A). A similar trend was found in ZO3, but it did not reach statistical significance. These preliminary results suggest that the combinatory effect of genetic factors in tandem with prenatal administration of FMT from ASD children has a significant impact on intestinal permeability, while the genetic factor itself is important only against the ASD microbiota background.

Analyses of the associations of bacterial genera that significantly differed in abundance in different groups with markers of intestinal permeability found that bacteria more abundant in WT animals or in mice prenatally exposed to FMT CTRL, particularly *Dubosiella*, *Butyricicoccus*, *Colidextribacter,* and *Roseburia,* had negative correlations with more than one marker, and opposite, more abundant taxa in *Shank3b^−/−^* animals, *Desulfovibrio* and *Turicibacter,* had positive correlations (Table 1). Interestingly, even if *Lactobacillus* had an increased abundance in *Shank3b^−/−^* mice, it still elicits a positive effect on gut permeability. 

## 3. Discussion

Recent data for ASD prevalence differ from 0.47% to 3.1% from country to country in Europe [51], with the Centers for Disease Control and Prevention announcing 2.8% for 2023 for the USA. Although the recorded prevalence depends on many factors, it is increasing worldwide, which applies pressure on scientists and clinicians to understand autism’s etiopathology and find not only objective biomarkers but also more effective and less time-consuming therapy. ASD is a polyetiological disorder characterized by significant genetic and environmental components, with the gut microbiota playing a role in their interaction [2]. This project aimed to investigate the involvement of each of these two factors and their interaction. Multiple preclinical studies have demonstrated that FMT from children with ASD into animals can induce ASD-like behaviors in the recipients [21,52,53]. In addition, it has been shown that FMT from healthy donors to ASD mice can improve ASD-related behaviors [54,55]. These findings suggest an important role of the microbiota in ASD development. Given the intensive nature of brain development during the prenatal period, the origins of ASD might be initiated before birth [46,47]. In addition, a recent study revealed that a mother’s microbiota is even more crucial for children’s neurodevelopment than its establishment within the first years [48]. Thus, we exposed female mice of the examined male offspring before gravidity to ASD and CTRL microbiota. The investigation of neurodevelopment, as hypothesized, found a delay in gait, proprioceptive, and vestibular functions in mice that received FMT ASD compared to those who received FMT CTRL, but only in WT mice. In adult *Shank3b^−/−^* males, impaired learning abilities and higher anxiety-like behavior after exposure to ASD microbiota was observed compared to male mice exposed to microbiota from neurotypical controls, which suggests the impact of microbiota on the comorbid symptoms of ASD. The genetic factor *Shank3b^−/−^* in these animals showed only a trend for a disagreeable effect on their social behavior in adulthood, but during adolescence, it had a strong negative influence on behavior, independently of the type of FMT. Moreover, FMT from ASD children had an ameliorating effect on the learning abilities of adolescent *Shank3b^−/−^* mice. This fact remains to be explained; however, a partial support of our finding was found in the study by Goo et al. (2020), who reported that FMT from WT mice to another genetic mouse model of ASD, Fmr1 KO mice, ameliorated ASD-like symptoms [56], suggesting the importance of microbiota even in genetic models of ASD. Beneficial effects of FMT from healthy humans on ASD-like behavior have been shown in another animal model of ASD—BTBR mice [55] as well.

We explored other impairments in people with ASD, and surrounding food intake behavior, we found that FMT ASD was associated with increased total food intake (1h) in adulthood, but only in WT males. This outcome suggests the major role of microbiota in regulating food intake and aligns with the results of a recent meta-analysis on the increased risk of obesity in children with ASD [57]. The correlation between the intake of and preferences for food was impaired after parental exposure to FMT ASD in WT animals, while in *Shank3b^−/−^* males, these relations were already impaired [58] and FMT ASD only restored them partially. It is known that any depletion of microbiota in mice, by antibiotic therapy or in germ-free mice, has many negative consequences [59]. Thus, probably in this case, any microbiota is better than its reduction. Significantly higher leptin concentrations [60,61] and their direct association with ASD in non-obese subjects exist [62]. In our study, the offspring of mice exposed to ASD microbiota had increased leptin concentrations in the plasma and higher food intake patterns, irrespective of Shank3 genotypes in lean mice. Leptin, as a mediator of the long-term regulation of energy balance, inhibits orexigenic (NPY/AgRP) and activates anorexigenic (POMC/CART) neurons in the arcuate hypothalamic nucleus, innervating HCRT LHA neurons [63]. Although we found decreased activity of HCRT LHA neurons after refeeding in animals of both genotypes prenatally exposed to ASD microbiota, the amount of total food intake during the 60 min-long sucrose test did not differ when compared to FMT CTRL-exposed animals. These discrepancies may indicate a shift in the balance between homeostatic and hedonic pathways regulating food intake in animals exposed to ASD microbiota. This is indicated by a disrupted correlation between sucrose solution preference and its intake, while the correlation between Fos-HCRT neuron activity and sucrose solution preference remains intact, similar to what has been observed with ethanol preference [64]. Interestingly, body weight in WT animals prenatally exposed to ASD microbiota increased on postnatal days 9 and 15, during which the development and maturation of leptin-sensitive projection pathways from the arcuate nucleus to the LHA is ongoing [65]. This body weight change might be associated with the altered correlations between food intake and preference found in adulthood. It has been shown that leptin concentrations correlate with *Cyanobacteria*, products of which decrease plasma triglycerides and BMI in obese people [66,67]. These bacteria had decreased abundance in our *Shank3b^−/−^* mice. *Parabacteroidetes* negatively correlated with leptin concentrations, which aligns with published research, where these bacteria alleviate obesity and obesity-related dysfunctions in mice [68].

Another important aspect of ASD is its gender disparity: boys are diagnosed with ASD approximately 4 times more often than girls [50]. One hypothesis of ASD development involves increased exposure to testosterone. The “fetal androgen theory” suggests that prenatal exposure to androgens may play a role in ASD development [69,70] and “the extreme male brain theory” builds on this idea by proposing that such exposure leads to a cognitive style more typical of males, which may underlie autistic traits [71]. A recent metanalysis regarding androgen concentrations in ASD patients has reported that free testosterone particularly may be elevated in individuals with ASD, with dehydroepiandrosterone being specifically elevated in ASD males [72]. Therefore, in this study, we evaluated circulating testosterone concentrations. Although we did not find its connection to ASD-like behavior or to the increased saccharose intake in mice as published before [73], we found higher circulating testosterone concentrations in WT male mice prenatally exposed to FMT ASD. In addition, a positive correlation between plasma testosterone concentrations and total food intake in WT mice after FMT ASDs of mice. Moreover, we found that plasma testosterone concentrations are associated with microbiota composition, consistent with findings from previous studies [74,75]. It is hypothesized that the microbiota is associated with ASD prevalence in boys either directly or through microbial metabolites and/or epigenetic factors capable of regulating host gene expression through DNA methylation and/or histone modification [76].

One already traditional explanation of frequent GI symptoms in children with ASD is inflammation and increased intestinal permeability, which was confirmed by increased fecal calprotectin [22] and serum zonulin levels [77]. Different bacteria are known to stimulate zonulin release, causing the loss of intestinal barrier function [78]. Solutes can pass the intestinal barrier using paracellular transport or through damaged epithelium. Paracellular transport has two tight-junction pathways, one for small-sized cations using pores formed by claudin, and the other, leak, for bigger-sized molecules, triggered by inflammatory cytokines such as TNF alpha [79]. In our study, claudin expression was significantly higher in the colon of *Shank3b^−/−^* mice after FMT from children with ASD compared to WT after the parental exposure to control microbiota, which might be a result of the combined effect of genetics and microbiota.

The leak pathway, as indicated by ZO1 expression, appears to be more strongly influenced by genetic factors, as in our experiment, *Shank3b^−/−^* mice with FMT ASD showed higher ZO1 levels compared to WT mice, not only after FMT ASD but also after FMT CTRL. Similarly, ZO3 expression was higher in *Shank3b^−/−^* mice with FMT from ASD donors. ZO3, along with ZO1 and ZO2, belongs to the zonula occludens family of scaffolding proteins, which interact with claudins, occluding, and F-actin to regulate tight junction permeability [80]. However, we did not observe significant changes in occludin expression. One of the possibilities is a compensatory mechanism of occludin downregulation to promote epithelial cell survival [79]. In this experiment, gut permeability markers showed negative correlations with several bacterial genera that were more abundant following FMT from NT children, such as *Dubosiella* and *Colidextribacter*, or in WT compared to *Shank3b^−/−^*, such as *Butyricicoccus, Dubosiella*, and *Roseburia*. *Lactobacillus* also decreased permeability marker expression consistently with other experiments, describing its beneficial probiotic effect on intestinal permeability [81,82]. As expected, a positive correlation was observed with increased in *Turicibacter* and *Desulfovibrio in Shank3b^−/−^ mice*, two genera previously linked to higher gut permeability and ASD [83]. Our findings are in line with recent data indicating that the disturbed microbiome of *Shank3b^−/−^* mice alters intestinal morphology and changes the expression of gut permeability markers [84]. Therefore, increasing evidence, including our own findings, highlights the potential and importance of prenatal and postnatal microbiota modulation in the prevention of ASD development [85,86]

## 4. Materials and Methods

### 4.1. Selection of Donors

Psychological and psychiatric examinations were performed at The Academic Research Center for Autism at the Institute of Physiology, Faculty of Medicine, Comenius University in Bratislava. Donors for ASD FMT were boys diagnosed with ASD using the “gold standard” of Autism Diagnostic Observation Schedule, Second Edition (ADOS-2) [87] and Autism Diagnostic Interview, Revised (ADI-R) [88], with a comparative score more than seven. Neurotypical children were recruited from the local population. The age of children with ASD and control donors was 5.61 ± 0.14 and 5.67 ± 0.16, correspondingly, and was not statistically different. Donors had not received any antibacterial or probiotic therapy for at least 2 months, maintained a BMI within normal ranges, and showed no symptoms of acute gastrointestinal issues or any other infections in the last 2 months. Written informed consent was obtained from parents of participating children. The protocol was approved by the Ethics Committee of the Comenius University Faculty of Medicine and the University Hospital, Bratislava, Slovakia (No. 79/2021). The study conformed to the code of ethics stated in the Declaration of Helsinki.

### 4.2. Experimental Animals

Pairs of heterozygous knockout B6.129-*Shank3*^tm2Gfng/J^ (*Shank3B^+/−^*) female and male mice were obtained from the Jackson Laboratory (017688, JAX Laboratory, Farmington, CT, USA) and transported to the animal facility at the Faculty of Medicine of the Comenius University in Bratislava. A harem scheme for breeding was used (1 male *Shank3B^+/−^* and 2 *Shank3B^+/−^* females per each cage). All pups were housed together with their mothers until the weaning period (PND 25). The genotyping of mouse pups was conducted by PCR using DNA extracted from ears during ear punching (approximately 3 mm) based on the Jackson Laboratory protocol at weaning (PND 25). Only male *Shank3B^−/−^* and WT mice were used in this study. Further, 12 WT and 7 *Shank3B^−/−^* mice were prenatally exposed to the FMT from neurotypical children (CTRL), and 5 WT and 6 *Shank3B^−/−^* mice underwent prenatal exposure to FMT from children with ASD. The mice were housed 4–5 per cage in the animal facility under controlled laboratory conditions (12:12 h light/dark cycle, temperature: 24  ±  2 °C, and humidity 55  ±  10%) with access to a standard pelleted diet and tap water ad libitum. All experimental procedures were approved by the Ethical Committee of the Institute of Pathophysiology (07/2017/SKU11016), Comenius University, Bratislava, and were conducted according to the European Union (EU) Directive 2010/63/EU and Slovak legislation. All efforts were made to minimize animal suffering and to reduce the number of animals used in this study.

### 4.3. Fecal Microbiota Transplant Preparation

Preparation. For the preparation of FMT, stool sample collection was performed at home by parents of neurotypical boys as well as boys with ASD into sterile flasks after a detailed explanation of the procedure. The samples were delivered to the laboratory within 2 h, and a fecal microbiota transplant was promptly prepared. Filtered frozen material in a final concentration of 10% glycerol was stored at −80 °C and used within 6 months [89]. To ensure biosafety, all fecal samples were tested for pathogens including bacteria, fungi, and parasites at the Institute of Microbiology, Faculty of Medicine, Comenius University in Bratislava. Additionally, intestinal inflammation was assessed by measuring calprotectin concentrations using CALPROLAB™ Calprotectin ELISA (ALP) (Calprolab, Lysaker, Norway), according to the instructions provided by the manufacturer. Fecal calprotectin concentrations below 50 μg/g of feces were considered within the normal range, based on the established criteria [90].

Fecal microbiota transplantation in mice. Prior to FMT, female heterozygous *Shank3B^+/−^* mice were treated with an antibiotic cocktail containing ampicillin, neomycin, metronidazole, and vancomycin (1 g/L, 1 g/L, 1 g/L and 500 mg/L, respectively) in drinking water ad libitum for 2 weeks according to previous literature [91,92,93]. After five days of recovery, 150 µL of fecal microbial transplant (either from ASD or neurotypical boys) was orally gavaged to heterozygous females (future mothers) for 3 consecutive days (Figure 1E) [89]. Before FMT, the donor material was completely thawed at room temperature. In this study, the transplanted microbiota was derived from a pooled mixture of stool samples from either 7 autistic or 6 neurotypical control children.

Consequently, breeding was initiated, and male *Shank3B^−/−^* and WT offspring were tested in a battery of tests to assess neurodevelopment (including physical and morphological landmarks of development), ASD-like behavior, and hedonic preference (Figure 1E). Biological samples were collected during and at the end of the experiment and stored at −80 °C until analyzed.

### 4.4. Assessment of Behavior in Mice

#### 4.4.1. Neurodevelopment

The physical and sensory–motor development of the pups was evaluated from postnatal day 1 (PND 1) until (PND 21) using a series of tests, as previously described [94]. Testing was conducted daily in the morning between 9:00 and 12:00 a.m.

Within physical and morphological landmarks, ear unfolding, eye opening, and incisor eruption were evaluated. The postnatal day when both ear pinnae were separated from the cranium, both eyes were open, and upper/lower incisors were observable was recorded.

Within reflexes, the following types of reflexes were evaluated:

**Forelimb grasp**: The paw of the pups was gently touched on the underside by a Q-tip. The presence of the grasping reflex was recorded.

**Forelimb placing**: The front paw of the pups was gently pressured against a perpendicular surface to record the presence of the paw adjusting reflex.

**Surface righting**: The pups were placed on their back and the amount of time the pups needed to fully turn over on belly was measured. Up to 60 s were allowed for the task, even if the task was not successfully fulfilled.

**Air righting** (from PND 4): The pups were held upside-down at about 25 cm above their cage and dropped onto a heavily padded surface. The ability of the pups to successfully right themselves while falling and land on all four paws was assessed.

**Auditory startle**: The presence of a startle reflex after the pups were exposed to an acoustic stimulus (fingers snapping) was evaluated.

**Tactile startle**: The presence of a startle reflex after the pups were exposed to a gentle puff of warm air was evaluated.

**Motor skills**: The pups were assessed using tests of negative geotaxis, gait and wire hanging:

**Negative geotaxis**: This test corresponds to the innate ability of rodents to detect gravitational stimuli. The pups were individually placed facing head down in the middle of a mesh-covered inclined surface (30° angle). The time required to turn around (180°) as an innate postural response to the detection of gravitational stimuli and climbing to the top of the mesh surface was measured with a maximum of 60 s.

**Gait and walking initiation**: The tested pups were placed in the center of 2 circles (with a radius of 5 cm and 15 cm, respectively). The time at which the pups crossed the outer border of the circle with all four limbs was measured. If the pups were unable to cross the outer circle in 60 s, they failed the test.

**Wire hanging** (from PND = 8). The first days when the animal, suspended by both paws on the rope (at a height of 30 cm), was able to hold on to it were recorded up to 60 s.

The **body weight** of the pups was also assessed daily following the neurodevelopmental milestones evaluation (from PND1 up to PND21) [94].

#### 4.4.2. Behavioral Phenotyping

WT and *Shank3^−/−^* offspring of mothers exposed to FMT underwent a complex behavioral phenotyping in both adolescence (PND 35-65) and adulthood (PND 90-130). Sociability, repetitive and anxiety-like behavior, and short- and long-term memory, were assessed. The behavioral phenotyping of mice was carried out using EthoVision XT 10 and 16.0 (Noldus Information Technology, Wageningen, Netherlands), and the following tests were implicated: open field test, elevated plus maze test, marble burying test, reciprocal social interaction test, Morris water maze test (with reversal learning) (Figure 1). Prior to behavioral testing, the animals were habituated to the testing room for a period of 1 h.

**Open field test.** The open field test was conducted in a square arena measuring 45 cm × 45 cm, which was virtually divided into two zones: the central zone and the border zone. Each mouse was placed at the center of the arena and allowed to explore freely for 10 min. Locomotor activity was assessed by measuring the total distance traveled. Additionally, the time spent in the central zone of open field arena was recorded as an indicator of anti-anxiety behavior [95].

**Elevated plus maze test.** The elevated plus maze consisted of four arms extending from the central platform (10 cm × 10 cm). Two opposing arms were open (45 cm × 10 cm), while the other two were enclosed by 40 cm high walls. The apparatus was elevated 50 cm above the floor on metal legs. Each mouse was placed at the end of an open arm, facing away from the maze, and allowed to explore freely for 5 min. Anti-anxiety-like behavior was assessed based on the time spent in the open arms [96].

**Marble burying test.** The marble burying test was performed in rectangular cages (40 × 25 cm × 25 cm) that were lined with sawdust bedding to a height of 5 cm. Twenty glass marbles (5 columns × 4 rows) were placed on the sawdust. The animals were placed in the center of the cage and allowed to freely explore the cage with marbles for 20 min. After the test was completed, the buried marbles were manually counted. A marble was considered buried when more than 2/3 of its volume was covered in sawdust [97].

**Reciprocal social interaction test.** Prior to reciprocal social interaction testing, animals were socially isolated for 24 h in individual maintenance cages (1 animal/cage). The social interaction test was performed in PhenoTyper cages with a square arena (45 cm × 45 cm, PhenoTyper, Noldus Information Technology, Wageningen, Netherlands) containing a 1 cm layer of sawdust bedding. Consequently, the mice were randomly paired with a socially unfamiliar mouse of the same sex, age, and genotype used as a social partner. The test lasted 10 min. Behavioral analysis was conducted by two blinded experimenters through manual scoring of recorded videos, where social interest and social disinterest initiated by the test animal towards the unknown social partner were monitored. Social interest was evaluated as cumulative time spent nose-to-nose, nose-anogenital, and side-sniffing, while indicators of social disinterest included self-grooming, digging, lying flat, freezing in contact, or actively avoiding the social partner [41,95,98].

**Morris water maze test.** The Morris water maze test was performed in a dark plastic circular pool with a diameter of 125 cm and a height of 60 cm. The pool was filled with water at a temperature of 25 ± 1 °C, made opaque with white nontoxic milk powder. The pool was virtually divided into four quadrants, each of which was marked with intra-maze geometric cues (circle, square, triangle, and star) within the maze wall to facilitate spatial learning and orientation. A circular platform (10 cm in diameter) was hidden in one of the four quadrants, 1 cm beneath the water surface. The location of the hidden platform was maintained in the same position throughout the four-day acquisition (initial learning) period. The mice were subjected to four trials per day, always starting from a distinct quadrant. During the acquisition phase of testing (days 1–4), the release positions were predetermined. One trial involved a swim that lasted up to 60 s, followed by a 30 s period of rest on the platform. An investigator carefully guided the mouse to climb on the platform if it failed to locate it within 60 s. Escape latency (the time required to locate the hidden platform) was calculated on a daily basis during the acquisition period to assess learning ability. On the fifth day of testing, a probe trial was performed. The platform was removed from the pool, and mice were allowed to swim for 60 s. The release position was in the quadrant that was opposite the platform-quadrant. The time spent in the platform quadrant was recorded as a measure of spatial reference memory [96,99].

Following the initial learning phase, the hidden platform was relocated to the opposite quadrant, and mice underwent an additional 4-day reversal learning phase to assess cognitive flexibility and adaptability to spatial changes. The testing procedure remained identical to the acquisition phase, with four trials per day, fixed release positions, and a maximum trial duration of 60 s. The primary measure of reversal learning was the escape latency to the new platform location. Similarly, as in the initial learning phase, at the end of the reversal learning phase, a reversal probe trial was conducted 24 h later [100].

### 4.5. Food Intake and Preference

Mice of both Shank3b genotypes prenatally exposed to CTRL or ASD microbiota (*n* = 4–12 per group) were housed individually in cages with standard equipment (bedding, a plastic house) and free access to standard chow pellets and two water bottles. The voluntary food intake and preference were studied in mice with free access to standard chow pellets and applied for 24 h sweet solution and sweet–fat solution tests. Food intake and preference induced by overnight fasting were studied in mice during the Novelty test and during the 60 min-lasting sweet solution test. Wash out periods for several days were between individual tests.

#### 4.5.1. Voluntary Food Intake and Preference in 24 H Lasting Sweet Solution and Sweet–Fat Solution Test

For this test, we followed a protocol earlier described [58]. Shortly, 2% of sucrose (sweet) solution and 2% of sucrose + 5% of fat (sweet–fat) solution in water were prepared. Following the habituation phase lasting for 24 h, water intake, applied solution intake, and consumed standard chow pellets were monitored in 24 h intervals (at 8:00 a.m.). Unlike the sweet solution, the sweet–fat solution was prepared and monitored in 12 h intervals for the prevention of instability due to the longer time interval at room temperature. To prevent any side preference in animals, the position of the tested bottles containing water and the tested solution was changed every 24 h. As described previously, the intake of individual compounds was calculated as consumed during 24 h (g) per animal’s body weight (g). The preference for sucrose solution and sucrose–fat solution to other tested compounds (standard chow pellets and water) or to water alone was calculated as the percentage of intake of appropriate solution intake (g) from the sum of all consumed compounds (g) or total liquid intake (g), respectively.

#### 4.5.2. Food Intake and Preference Induced by Overnight Fasting During the Novelty Test and 60 Min Lasting Sweet Solution Test

As described previously [58], standard chow pellets with semi-hard edam cheese, fresh apple, durable salami, and chocolate bar were offered to overnight-fasted mice with free access to water for 3 h. The total food intake was calculated as the sum of the weights (g) of all five consumed items per animal’s body weight (g) during the first 60 min (1 h) and during the 180 min period (3 h). Food intake of tested items was calculated as the consumption of five food items (g) per animal body weight (g) during the first 1 h and the 3 h period. The preference for a tested item was calculated as the percentage of the weight of an appropriate item (g) from the sum of the weights of all consumed items (g) per animal during the first 1 h and the 3 h period. The correlation analysis between the amount of a consumed item and its individual preference in mice was performed for the first 1 h. The correlation analysis between the individual total food intake and individual plasma testosterone and leptin level in mice was performed for the first 1 h and for the 3 h period.

As described previously [58], standard chow pellets and 2% sucrose solution were offered to overnight fasted mice with free access to water for 60 min. The intake was calculated as the weight of consumed food or liquid during 60 min (g) per animal’s body weight (g). The preference for tested compounds was calculated as the percentage of the weight of a consumed tested compound (g) from the sum of the weights of all consumed compounds (g). The preference for 2% sucrose solution compared to water was calculated as the percentage of the weight of 2% sucrose solution (g) from the total liquid intake (g) of animals. Finally, the correlation analysis between the individual total intake of all compounds and sucrose preference prior to water with individual plasma testosterone, leptin, and NPY levels was performed in mice.

### 4.6. Blood Analysis

Blood collection was performed from the retro-orbital sinus into the EDTA collection tube (K3 EDTA, Microvette, Sarstedt, Nümbrecht, Germany). The blood was centrifuged for 10 min at 1600× *g* and 4 °C, and the plasma was stored at −80 °C until further analysis. Besides measuring the concentrations of leptin (SIGMA-ALDRICH, St. Louis, MO, USA) and neuropeptide Y (EMD Millipore Corporation, Billerica, MA, USA), the plasma samples were used to measure the testosterone level (DRG Instruments GmbH, Marburg, Germany), according to the manufacturer instructions.

### 4.7. Fos and Fos-HCRT Immunohistochemistry

The immunohistochemical study was performed 90 min after the beginning of the sweet solution test, which was 30 min after the withdrawal of standard chow pellets and sucrose solution. At the end of the experiment, mice were deeply anesthetized with sodium pentobarbital (50 mg/kg, i.p.) and perfused transcardially with 0.1 M phosphate buffer (PB, pH 7.4) containing 4% paraformaldehyde. The immunohistochemical study was performed 90 min after the beginning of the sweet solution test, which means 30 min after the withdrawal of standard chow pellets and sucrose solution. At the end of the experiment, mice were deeply anesthetized with sodium pentobarbital (50 mg/kg, i.p.) and perfused transcardially with 0.1 M phosphate buffer (PB, pH 7.4) containing 4% paraformaldehyde. The mouse brains were removed and Fos and Fos-HCRT immunoreactivity was determined as described before [101]. For the immunohistochemical study, rabbit c-Fos monoclonal antibody (1:2000; #2250S; Cell Signaling Technology, Inc., Danvers, MA, USA) and HCRT rabbit monoclonal antibody (1:100, #16743S; Cell Signaling Technology, Inc., Danvers, MA, USA) was used.

The HCRT and Fos-HCRT immunoreactive neurons were counted separately on each side of the appropriate coronal sections (*n* = 3 sections per mouse) within the LHA (from bregma −1.22 mm to bregma −1.46 mm) according to the mouse brain atlas [102]. Quantitative assessment of the immunostained cells was performed manually under light microscope ZEISS Axio scope A1 (Carl Zeiss Microscopy, LLC, White Plains, NY (USA)) and digital camera (AxioCam Erc 5s ZEISS, White Plains, NY (USA)). The percentage of activated HCRT neurons was calculated by formula: 100 × (amount of HCRT neurons displaying Fos signal/total HCRT immunolabeled perikarya). It was expressed unilaterally per one section/animal.

### 4.8. RT-PCR

We performed RT-PCR of the gut permeability markers in the colon of experimental animals to analyze the prenatal impact of FMT on the intestines of Shank3b mice. The total RNA was isolated using a phenol–chloroform extraction method with TRIzol^®^Reagent (Invitrogen, Carlsbad, CA, USA), according to the manufacturer’s protocol. The concentrations were measured by NanoDrop 2000 (Thermo Fisher Scientific, Waltham, MA, USA). Reverse transcription of the RNA was performed using the High-Capacity cDNA Reverse Transcription Kit (Thermo Fisher Scientific, Waltham, MA, USA), according to the manufacturer’s protocol. Semi-quantitative real-time PCR was performed in a total volume of 25 μL containing 30 ng of template cDNA mixed with 12.5 μL FastStart Universal SYBR Green Master Rox (Roche Diagnostics, Mannheim, Germany), 1 μL specific primer pair set, and water. The sequences of the specific primers used were as follows: zonula occludens 1 (ZO1-F, MR AAGAAAAAGAATGCACAGAGTTGTT, a ZO1-R MR, GAAATCGTGCTGATGTGCCA), ZO3 (ZO3-F, GATTGTTTCCAGGCCCCTCC, a ZO3-R, ACCAGCACGGACTTTAGTGG), Claudin5 (claud5-F MR, GTTAAGGCACGGGTAGCACT, a claud5-R MR, GTACTTCTGTGACACCGGCA), occludin (occlud-F T, CCTCCAATGGCAAAGTGAAT, an occlud-R T, CTCCCCACCTGTCGTGTAGT). Each sample was analyzed using the QuantStudio™ 5 Real-Time PCR System (Applied Biosystems, Waltham, MA, USA) under the following thermal cycling conditions: an initial step of 2 min at 50 °C, followed by 10 min at 95 °C, and then 40 cycles of 15 s at 95 °C and 1 min at 60 °C. Gene expression changes were determined using the Livak (2^–ΔΔCt^) method [103], with GAPDH serving as the housekeeping gene. To verify the specificity of the amplified products, a melting curve analysis was conducted.

### 4.9. Microbiota Analysis

DNA was extracted from frozen stool samples using a commercial extraction system (QIAamp DNA Fast Stool Mini Kit, Qiagen, Hilden, Germany), according to the manufacturer’s instructions. The DNA concentration was determined using the NanoDrop 2000 Spectrophotometer (ThermoFisher Scientific, Waltham, MA, USA). High-throughput sequencing was performed for the DNA libraries using the PCR amplification of the V1–V3 region of 16S rRNA. In this process, 3–50 ng of total input DNA in a 20 µL volume PCR reaction was amplified with 4 µL of 5x HOT FIREPol Blend Master Mix (Solis BioDyne, Tartu, Estonia), 0.4 µL (10 µM) of each primer (final concentration 0.2 µM), and milli-pore water. The PCR conditions were as follows: initial denaturation 95 °C/15 min, cycling 25× (95 °C /20 s, 60 °C/30 s, 72 °C/2 min), final polymerization 72 °C/10 min. Amplicons were column-purified (Zymo DNA Clean and Concentrator-5, Zymo Research, Irvine, CA, USA) according to standard protocols and quantified fluorometrically with the Qubit™ dsDNA HS Assay Kit (Thermo Fisher Scientific, Waltham, MA, USA). Amplicon sequences were fragmented by a transposon-based approach (Nextera XT, Illumina San Diego, CA, USA), and low-cycle PCR and mutual indexing of the fragments was performed. Fragment size selection and purification with 1.8x AMPure XP beads yielded final DNA libraries that were verified using the Agilent 2100 Bioanalyzer (Agilent Technologies, Waldbronn, Germany) and quantified using the Qubit 2.0 Fluorometer (Thermo Fisher Scientific, Waltham, MA, USA). The 4 nM pool of libraries was further diluted to 10pM and sequenced on the Illumina MiSeq platform (Illumina, San Diego, CA, USA) with 2 × 300 bp paired-end sequencing at the Comenius University Science Park (Bratislava, Slovakia). Library sequence data were quality checked using FastQC, Andrews, 2010, a quality control tool for high-throughput sequence data available online at http://www.bioinformatics.babraham.ac.uk/projects/fastqc (accessed on 20 September 2024). Further data processing, including trimming, 16S analysis, and visualization, was performed with Geneious (Biomatters Ltd., Auckland, New Zealand) (https://www.geneious.com/ accessed on 23 September 2024). For visualizing and clustering multivariate data, principal component analysis using (PCA) ClustVis (https://biit.cs.ut.ee/clustvis/, accessed on 24 September 2024) [104] and MicrobiomeAnalyst [105,106] (https://www.microbiomeanalyst.ca/MicrobiomeAnalyst/ModuleView.xhtml, accessed on 10 December 2024) was applied. For diversity calculations, the Bray–Curtis Index and Permanova test were used. Real-time PCR was performed using FastStart Universal SYBR Green Master (Rox) (Roche), according to the manufacturer’s instructions. Primers were obtained from the literature (Table S1) [20,107,108,109,110] and ordered from Eurofins MWG Operon, Ebersberg, Germany. 

### 4.10. Statistical Analysis

Normality was evaluated by the Shapiro–Wilk test followed by a t-test or Mann–Whitney test for comparing two, or one-way, ANOVA parametric, or non-parametric, tests for comparing more groups. In this case, Tukey’s or Dunn’s correction test was applied. Based on distribution, Pearson or Spearman tests were used for correlations. For statistical comparison, data obtained from food intake and preference studies and the means of percentage of activated HCRT neurons in the LHA two-way analysis of variance (ANOVA) for the factors of genotype and prenatally applied FMT were used with subsequent post hoc Tukey’s tests. The Pearson’s correlation coefficient (r) was analyzed in the Novelty test between the individual amount of a consumed item and its individual preference during the first 1 h, and between the individual total food intake and individual plasma testosterone and leptin level in mice during the first 1 h and the 3 h period; in the 60 min-lasting sucrose test between the individual total intake of all compounds as well as sucrose preference prior to water with individual plasma testosterone, leptin, and NPY level; in the 60 min-lasting sucrose test between the individual sucrose solution preference and its intake from total intake of all compounds; and between the individual sucrose solution preference from total intake of all compounds and percentage of Fos-HCRT neurons in LHA. The positive and negative r values indicated direct and indirect correlations between values, respectively. The r = 0 means no correlation between variables, r < 0.35 weak, correlation, r = 0.36 to 0.67 moderate correlation, r = 0.68 to 0.89 strong correlation, and r > 0.90 very strong correlation. *p* < 0.05 was considered statistically significant. For hedonic behavior software Statistica 7.0, StatSoft was used, and for markers of intestinal permeability and the activity of astrocytes, GraphPad Prism 8.4.3 was employed.

#### Limitations

One major limitation of our study was the challenge in obtaining a sufficient number of offspring with the desired combination of sex, *Shank3b* genotype, and FMT exposure. Additionally, while animal models do not fully replicate the behavioral characteristics of human ASD, they offer important advantages. Particularly, animal models allow for the examination of mechanisms like gut permeability and brain center activation, which cannot be ethically investigated in human volunteers. Although ASD is more often found in males, future experiments could expand to female offspring for universal application of investigated mechanisms.

## 5. Conclusions

Our study confirms that genetic circumstances influenced by environmental factors contribute to the development of ASD. To our knowledge, this is the first study to examine the postnatal effect of parental exposure to a foreign microbiome in combination with genetic background for autism. Wild-type male offspring whose parents were exposed to FMT from children with ASD showed delayed neurodevelopment. When combined, the *Shank3b^−/−^* genetic mutation and FMT from ASD donors led to ASD-like behavior in adult offspring, along with increased markers of gut permeability. Prenatal exposure to the gut microbiota from children with ASD led to impaired postnatal regulation of food intake in *Shank3b^−/−^* males. In addition, parental exposure to the microbiota from children with ASD resulted in elevated plasma leptin concentrations, higher food intake and body weight, and suppression of HCRT neurons in the LHA in the offspring. While this study offers new insights into ASD pathogenesis, further research is necessary to confirm these findings.

## Figures and Tables

**Table 1:** Correlation of the expression of gut permeability markers ZO1, ZO3, occluding, and claudin in the colon with selected bacterial genera with significantly different abundance in WT and *Shank3b^−/−^* mice exposed to FMT from ASD children and neurotypical controls.

	ZO3	ZO1	Claudin	Occludin
Dubosiella	−0.44	−0.37	−0.42	−0.33
	*p* < 0.05	*p* < 0.05	*p* < 0.05	*p* < 0.05
Butyricicoccus		−0.47	−0.42	−0.36
	NS	*p* < 0.01	*p* < 0.05	*p* < 0.05
Colidextribacter	−0.47	−0.45	−0.41	
	*p* < 0.05	*p* < 0.01	*p* < 0.05	NS
Roseburia	−0.50	−0.48	−0.54	
	*p* < 0.01	*p* < 0.01	*p* < 0.01	NS
Lachnoclostridium	−0.44	−0.37	−0.39	−0.36
	*p* < 0.05	*p* < 0.05	*p* < 0.05	*p* < 0.05
Lactobacillus				−0.33
	NS	NS	NS	*p* < 0.05
Desulfovibrio	0.55	0.40	0.50	
	*p* < 0.01	*p* < 0.05	*p* < 0.01	NS
Turicibacter	0.38		0.35	
	*p* < 0.05	NS	*p* < 0.05	NS
Parabacteroides		0.45		
	NS	*p* < 0.01	NS	NS

## Data Availability

Data is contained within the article and Supplementary Materials.

## Supplementary Materials

The following supporting information can be downloaded at https://www.mdpi.com/article/10.3390/ijms26135927/s1. References [107,108,109,110] are cited in the Supplementary Material.
