# Supplementary material for: The Effect of Parental Faecal Microbiome Transplantation from Children with Autism Spectrum Disorder on Behavior and Gastrointestinal Manifestations in the Male Offspring of Shank3 Mice
Source: Int J Mol Sci. 2025 Jun 20;26(13):5927. doi: 10.3390/ijms26135927 (PMC12249950; doi:10.3390/ijms26135927)
Supplement: Supplementary file 1 [file ijms-26-05927-s001.zip › ijms-3680346-supplementary.pdf]

Supplementary material to "The effect of parental faecal microbiome transplantation from children with autism spectrum disorder on behavior and gastrointestinal manifestations in the male offspring of *Shank3* mice" by Borbélyová et al.

Neurodevelopmental testing

Besides the main findings, several parameters presented with statistically significant effects likely attributable to methodological variability within the final days of neurodevelopmental testing. In the walking task, the groups differed in time required to pass the test at PND16 [F(3,26)=2.8, p<0.05], PND18 [F(3,26)=5.7, p<0.01] and PND20 [F(3,26)=4.1, p<0.05, Figure S1A]. At PND16, this effect was only nominal and did not translate into significant between-group effects in post-hoc comparison. At PND18, the effect was comprised of WT ASD mice, that required longer time to perform the task (27.2 ± 9.8 s, p<0.01) than the rest of the groups (WT CTRL: 7.3 ± 2.1 s, *Shank3*$^{-/-}$ CTRL: 5.8 ± 1.6 s, *Shank3*$^{-/-}$ ASD: 6.2 ± 1.4 s). At PND20, the effect was fully explained by the *Shank3*$^{-/-}$ CTRL mice, which required the least time to complete the task (6.5 ± 2.1 s, p<0.05) among all groups (WT CTRL: 10.1 ± 4.7 s, WT ASD: 36.5 ± 12.9 s, *Shank3*$^{-/-}$ ASD: 12.2 ± 3 s). Additionally, no significant differences were observed between the groups in the first time to perform the walk milestone [F(3,26)=1.3, p=0.28, Figure S1B]. In surface righting, the animal groups different in time required to complete the test at PND10 [F(3,26)=4.1, p<0.05, Figure S1C]. The Bonferroni-corrected post-hoc test showed this effect was caused exclusively by the *Shank3*$^{-/-}$ CTRL group, which required the most time to pass the test (4.1 ± 1.1 s, p<0.05) among all the groups (WT CTRL: 1.3 ± 0.2 s, WT ASD: 1.5 ± 0.6 s, *Shank3*$^{-/-}$ ASD: 1.7 ± 0.5 s). No statistical differences between the groups were observed in the first time to pass the surface righting [F(3,26)=0.85, p=0.48, Figure S1D]. The time required to turn within the negative geotaxis task presented with high variability, resulting in no statistical difference among the groups at any PND (p>0.05, Figure S1E). Similarly, no group differences were observed in the first time to turn successfully within the negative geotaxis task [F(3,26)=2.4, p=0.09, Figure S1F].

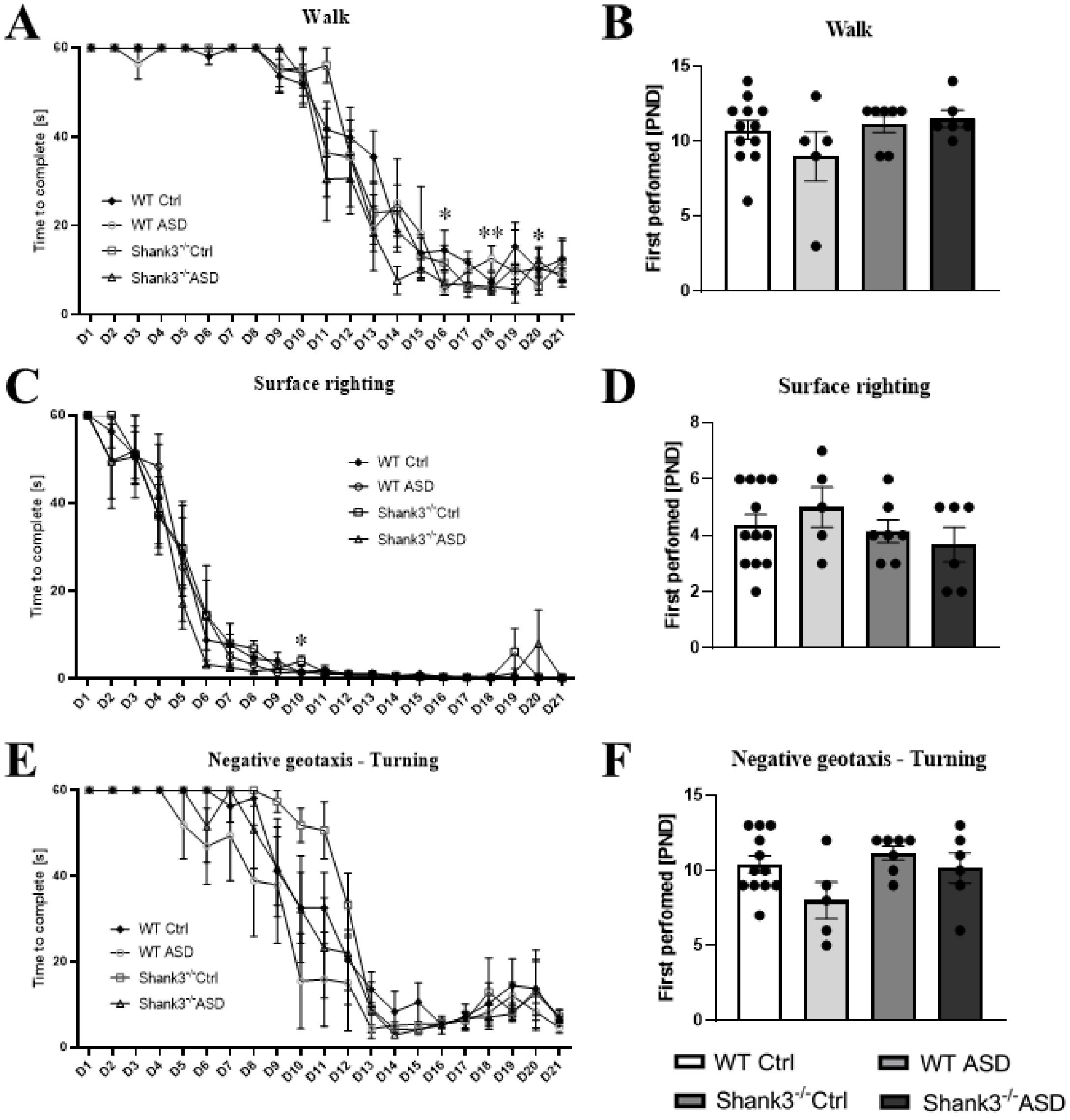

Figure S1: Neurodevelopmental milestones. A. Walk of mice during the testing period (D16, *=omnibus effect; D18, **=WT ASD > all; D20, *=*Shank3*$^{-/-}$ Ctrl < all) and B. the first day of walk in animals. C. Surface righting in mice during the testing period (*=*Shank3*$^{-/-}$ Ctrl > all) and D. the first day of surface righting in animals. E. Negative geotaxis in mice during the testing period and F. the first day of surface righting in animals. ASD – mice prenatally exposed to fecal microbiota transplantation (FMT) from children with ASD, Ctrl - mice prenatally exposed to FMT from neurotypical children, mean±SEM, * p<0.05, ** p<0.01

No significant differences between the groups were observed in the first time the ears of the animals unfolded [F(3,26)=1.3, *p*=0.28, Figure S2A], the ears twitched [F(3,26)=0.53, *p*=0.67, Figure S2B], the animals were able to right themselves in the air [F(3,26)=1.8, *p*=0.18, Figure S2C], their incisors erupted [F(3,26)=1.7, *p*=0.2, Figure S2D], they were able to grasp with their front paws [F(3,26)=2.7, *p*=0.06, Figure S2E], or adjust the front paws to a perpendicular surface [F(3,26)=2.1, *p*=0.12, Figure S2F], nor when the auditory [F(3,26)=0.99, *p*=0.41, Figure S2G], or tactile startle reflex developed [F(3,26)=2.1, *p*=0.13, Figure S2H]. However, the groups differed in the

first time their eyes opened [F(3,26)=5.2, *p*<0.01, Figure S2I]. This effect was explained by the WT ASD group, which opened their eyes earlier (12.2 ± 0.4 PND, *p*<0.05) than the WT CTRL group (13.5 ± 0.3 PND).

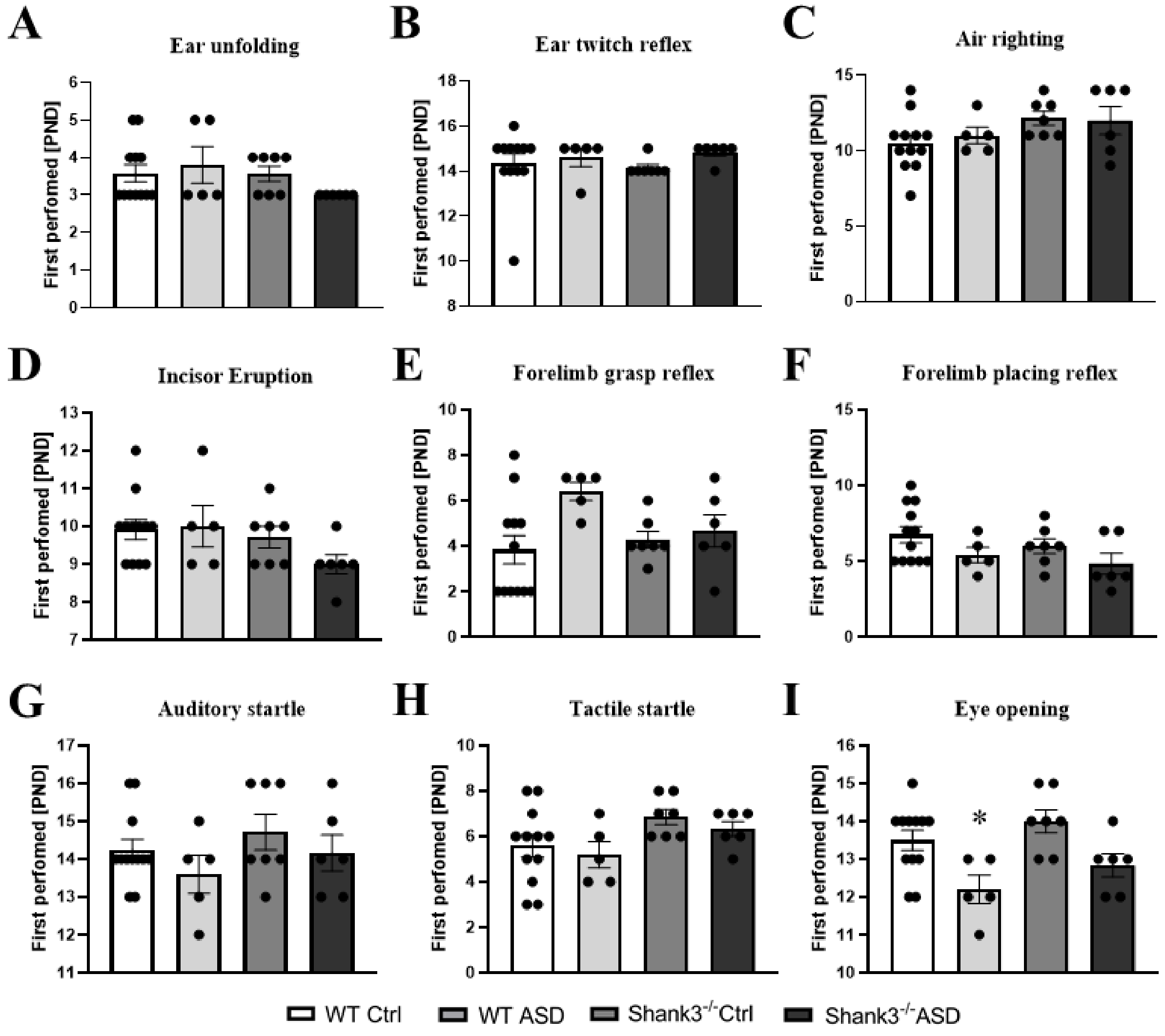

Figure S2: Neurodevelopmental milestones. First day of A. Ear unfolding B. Ear twitch reflex C. Air righting reflex D. Incisor eruption E. Forelimb grasp reflex F. Forelimb placing reflex. G. Auditory startle. H. Tactile startle. I. Eye opening (*=WT ASD < WT Ctrl). ASD –mice prenatally exposed to fecal microbiota transplantation (FMT) from children with ASD, Ctrl - mice prenatally exposed to FMT from neurotypical children. mean±SEM, *=p<0.05.

Behavioral testing in adolescence

Beside the main findings from the behavioral testing in adolescence, the rest of the behavioral parameters showed no difference between the groups. These included the analysis of locomotor activity [$F(3,26)=2.6$, $p=0.07$, Figure S3A], explorative behavior [$F(3,26)=1.6$, $p=0.22$, Figure S3B], repetitive behaviors [$F(3,26)=4.1$, $p=0.16$, Figure S3C], anxiety-like behavior measured via time spent in the center zone of the Open field test [$F(3,26)=2.4$, $p=0.09$, Figure S3D], as well as measured via time spent in open arms of the Elevated plus maze [$F(3,26)=0.96$, $p=0.42$, Figure S3E], marble burying [$F(3,26)=1.5$, $p=0.24$, Figure S3F], social disinterest [$F(3,26)=0.31$, $p=0.82$, Figure S3G], learning [$F(3,26)=1.6$, $p=0.21$, Figure S3H], and cognitive flexibility [$F(3,26)=2.26$, $p=0.11$, Figure S3I]. The only additional behavioral parameter which produced a significant group difference in adolescence was the training part of the reversal learning phase of the Morris water maze. This difference was observable on day 1 [$F(3,26)=6.3$, $p<0.01$] and on day 2 [$F(3,26)=3.2$, $p<0.05$, Figure S3G]. On day 1, the effect was explained by the *Shank3*$^{-/-}$ CTRL mice, which exhibited the longest time locating the hidden platform (50.4 ± 3.1 s, $p<0.01$) among the rest of the groups (WT CTRL: 34.7 ± 3.4 s, WT ASD: 26.8 ± 4 s, *Shank3*$^{-/-}$ ASD: 31.4 ± 4.2 s). On day 2, the effect was explained by the WT ASD mice, which showed the shortest time locating the hidden platform (13.7 ± 4.7 s, $p<0.05$) of all the groups (WT CTRL: 34.9 ± 5 s, *Shank3*$^{-/-}$ CTRL: 39.7 ± 5.4 s, *Shank3*$^{-/-}$ ASD: 31.3 ± 5.9 s).

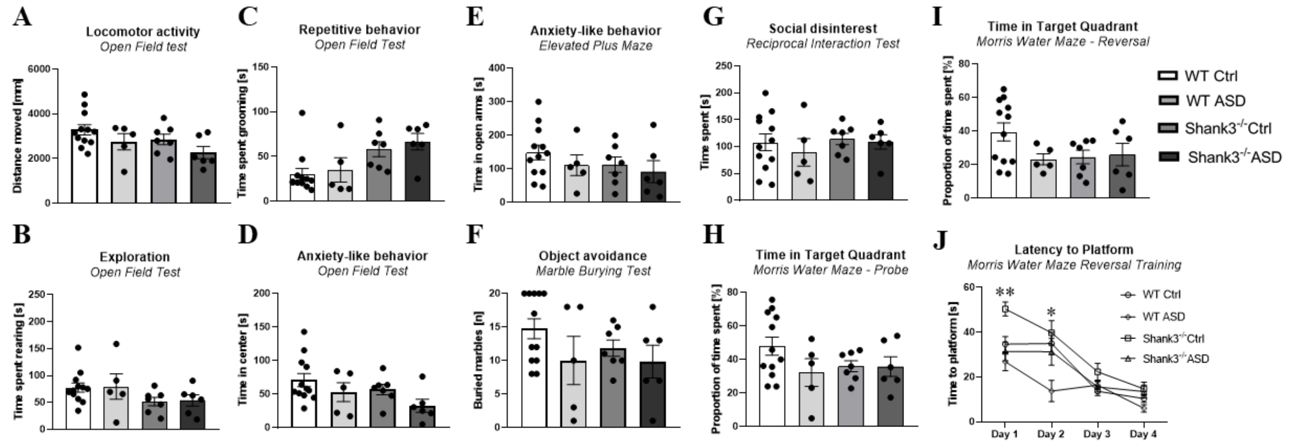

Figure S3: Behavioral testing in adolescence. A. Locomotor activity. B. Exploratory behavior. C. Repetitive self-grooming behavior. D-E. Anxiety-like behavior. F. Object avoidance behavior. G. Social disinterest. H. Reference memory. I. Reference memory in the Reversal Morris water maze test. J. Short-term spatial memory during reversal learning (D1, **=*Shank3*$^{-/-}$ Ctrl > all; D2, *=WT ASD < all). ASD –mice prenatally exposed to fecal microbiota transplantation (FMT) from children with ASD, Ctrl - mice prenatally exposed to FMT from neurotypical children. D=postnatal day. mean±SEM, *=p<0.05, **=p<0.01.

Behavioral testing in adulthood

Beside the main findings from the behavioral testing in adulthood, the rest of the behavioral parameters showed no difference between the groups. These included the analysis of locomotor activity [$F(3,26)=2.2$, $p=0.1$, Figure S4A], explorative behavior [$F(3,26)=2.3$, $p=0.09$, Figure S4B], repetitive behavior [$F(3,26)=4.1$, $p=0.17$, Figure S4C], anxiety-like behavior measured via the time spent in the center zone of the Open field test [$F(3,26)=1.5$, $p=0.24$, Figure S4D], marble burying [$F(3,26)=0.77$, $p=0.52$, Figure S4E], social disinterest [$F(3,26)=1.6$, $p=0.23$, Figure S4F], learning [$F(3,26)=2.5$, $p=0.08$, Figure S4G], and cognitive flexibility [$F(3,26)=1.64$, $p=0.21$, Figure S4H]. Similar to the behavioral testing during adolescence, the only additional behavioral parameter which produced a significant group difference in adulthood was the training part of the reversal learning phase of the Morris water maze. Once again, we observed a similar finding – the groups differed only on day 1 [$F(3,26)=3.3$, $p<0.05$] and day 2 of the training phase [$F(3,26)=2.9$, $p<0.05$, Figure S4I]. Importantly, though, neither of these effects were large enough to produce a between-group difference in a Bonferroni-corrected post-hoc analysis.

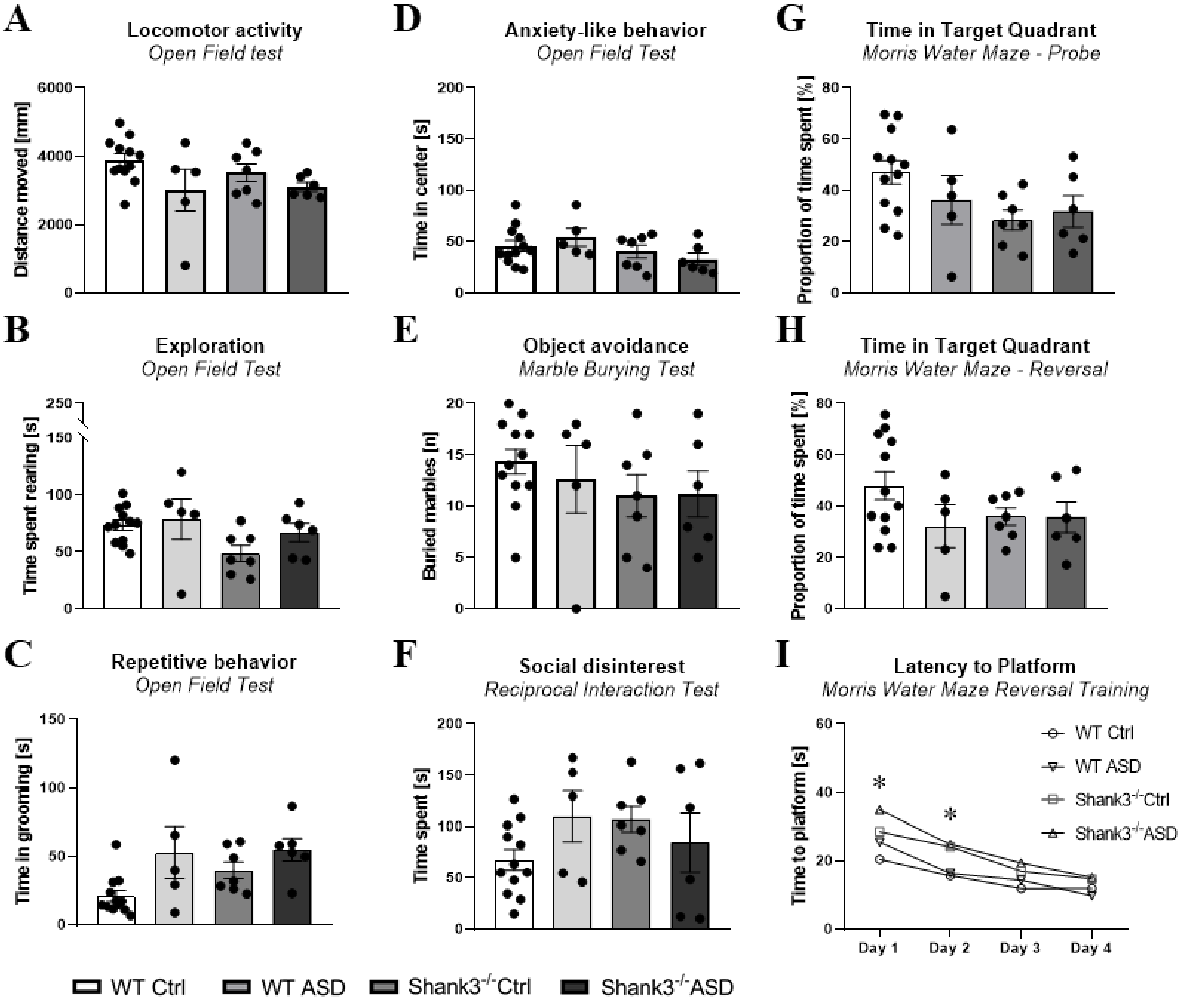

Figure S4: Behavioral testing in adulthood. A. Locomotor activity. B. Exploratory behavior. C. Repetitive self-grooming behavior. D. Anxiety-like behavior. E. Object avoidance behavior. F. Social disinterest. G. Reference memory. H. Reference memory in Reversal Morris water maze test. I. Short-term spatial memory during reversal learning (D1, D2, *=omnibus effect). ASD –mice prenatally exposed to fecal microbiota transplantation (FMT) from children with ASD, Ctrl - mice prenatally exposed to FMT from neurotypical children. mean±SEM, *=p<0.05.

Besides direct findings in differences of the gut microbiota in male offspring using 16sRNA sequencing, during the experiment we monitored the transplantat and microbiota transfer by Real-Time PCR using following primers (Table S1).

Table S1. Primers used for Real-Time PCR for continual control of fecal microbiota transplantation.

Primer Name	Primer Sequence (5´ - 3´)	Amplicon Length	Target	Positive Control	Reference
Bact934F	GGARCATGTGGTTTATTCGATGAT	60	Bacteroidetes	Bacteroides fragilis	Guo et al., 2008
Bact1060R	AGCTGACGACAACCATGCAG	60	Bacteroidetes	Bacteroides fragilis	Guo et al., 2008
Firm934F	GGAGYATGTGGTTTAATTCGAAGCA	60	Firmicutes	Clostridia	Guo et al., 2008
Firm1060R	AGCTGACGACAACCATGCAC	60	Firmicutes	Clostridia	Guo et al., 2008
F-bifido	CGCGTCYGGTGTGAAAG	60	Bifidobacterium spp.	Bifidobacterium longum	Delroisse et al., 2008
R-bifido	CCCCACATCCAGCATCCA	60	Bifidobacterium spp.	Bifidobacterium longum	Delroisse et al., 2008
Lacto-F	AGCAGTAGGGAATCTTCCA	60	Lactobacillus spp.	Lactobacillus rhamnosus	Wang et al., 2011
Lacto-R	CACCGCTACACATGGAG	60	Lactobacillus spp.	Lactobacillus rhamnosus	Wang et al., 2011
Prev F	CACRGTAAACGATGGATGCC	21	Prevotella	Prevotella copri	Bekele et al., 2010
Prev R	GGTCGGGTTGCAGACC	17	Prevotella	Prevotella copri	Bekele et al., 2010
ITS1F	CTTGGTCATTTAGAGGAAGTAA	22	Fungi	Candida albicans	Tang et al., 2015
ITS2R	GCTGCGTTCTTCATCGATGC	20	Fungi	Candida albicans	Tang et al., 2015

Guo X, Xia X, Tang R, Zhou J, Zhao H, Wang K. Development of a real-time PCR method for Firmicutes and Bacteroidetes in faeces and its application to quantify intestinal population of obese and lean pigs. Lett Appl Microbiol. 2008 Nov;47(5):367-73. doi: 10.1111/j.1472-765X.2008.02408.x. PMID: 19146523.

Delroisse JM, Boulvin AL, Parmentier I, Dauphin RD, Vandenbol M, Portetelle D. Quantification of Bifidobacterium spp. and Lactobacillus spp. in rat fecal samples by realtime PCR. Microbiol Res. 2008;163(6):663-70. doi: 10.1016/j.micres.2006.09.004. PMID: 19216105.

Bekele AZ, Koike S, Kobayashi Y. Genetic diversity and diet specificity of ruminal Prevotella revealed by 16S rRNA gene-based analysis. FEMS Microbiol Lett. 2010 Apr;305(1):49-57. doi: 10.1111/j.1574-6968.2010.01911.x. Epub 2010 Jan 27. PMID: 20158525.

Tang J, Iliev ID, Brown J, Underhill DM, Funari VA. Mycobiome: Approaches to analysis of intestinal fungi. J Immunol Methods. 2015 Jun;421:112-121. doi: 10.1016/j.jim.2015.04.004. Epub 2015 Apr 17. PMID: 25891793; PMCID: PMC4451377.

Wang L, Christophersen CT, Sorich MJ, Gerber JP, Angley MT, Conlon MA. Low relative abundances of the mucolytic bacterium Akkermansia muciniphila and Bifidobacterium spp. in feces of children with autism. Appl Environ Microbiol. 2011 Sep;77(18):6718-21. doi: 10.1128/AEM.05212-11. Epub 2011 Jul 22. PMID: 21784919; PMCID: PMC3187122.
